# Long-Term Physical Activity Effectively Reduces the Consumption of Antihypertensive Drugs: A Randomized Controlled Trial

**DOI:** 10.3390/jcdd10070285

**Published:** 2023-07-03

**Authors:** Alessandra di Cagno, Giovanni Fiorilli, Andrea Buonsenso, Giulia Di Martino, Marco Centorbi, Antonella Angiolillo, Giuseppe Calcagno, Klara Komici, Alfonso Di Costanzo

**Affiliations:** 1 Department of Movement, Human and Health Sciences, University of Rome “Foro Italico”, 00135 Rome, Italy; alessandra.dicagno@uniroma4.it; 2 Department of Medicine and Health Sciences, University of Molise, 86100 Campobasso, Italy; fiorilli@unimol.it (G.F.); andrea.buonsenso@unimol.it (A.B.); giulia.dimartino21@gmail.com (G.D.M.); marco.centorbi@hotmail.it (M.C.); 3 Centre for Research and Training in Medicine of Aging, Department of Medicine and Health Science “V. Tiberio”, University of Molise, 86100 Campobasso, Italy; angiolillo@unimol.it (A.A.); klara.komici@unimol.it (K.K.); alfonso.dicostanzo@unimol.it (A.D.C.)

**Keywords:** blood pressure, exercise, antihypertensive drug load

## Abstract

Background: Although physical activity (PA) has been shown to enhance hypertension control, the impact of exercise on the potential decrease of the use of antihypertensive medications remains inadequately researched. Aim: The aim was to assess the impact of a two-year PA on the medication requirements of individuals with hypertension. Methods: A clinical trial was conducted, involving 130 participants with essential hypertension who took at least one antihypertensive medication. Participants were randomly assigned to either a control group (CG *n* = 65) or an experimental group (EG *n* = 65) that underwent a 24-month supervised PA program based on a combination of aerobic and resistance training. The antihypertensive drug load for each participant was determined by adding the ratios of the prescribed daily dose (PDD) to the defined daily dose (DDD) for all antihypertensive medications taken by the participants. The outcome measures were evaluated at 0, 6, 12, 18, and 24 months. Results: A total of 76 participants completed the 24-month assessment, and RM-ANOVA revealed a significantly lower antihypertensive drug load in the EG compared to the CG at 18 (*p* < 0.017) and 24 months (*p* < 0.003). Conclusion: A long-term PA program can decrease the antihypertensive drug load in older adults with essential hypertension. The trend of improvement regarding the EG drug load intake and the trend of CG drug load increase, although not significant over time, results in a significant difference between the groups at 18 months and an even greater difference at 24 months. This trend certifies the protective value of PA against the aging process and its related health risk factors.

## 1. Introduction

Arterial hypertension (HTN) is a prevalent risk factor for major cardiovascular diseases (CVD), and its management through various classes of antihypertensive treatments has demonstrated significant benefits in terms of HTN control and outcomes [1]. In recent years, life expectancy has risen considerably, resulting in a higher proportion of elderly individuals within the general population. It is noteworthy that approximately 70% of older adults are afflicted with hypertension, making it one of the most common and treatable conditions [2]. Despite advancements in hypertension management, individuals with HTN remain at an elevated risk for cardiovascular mortality, and this risk is particularly pronounced in the elderly population. Older adults with HTN also experience a range of negative consequences, including functional decline, disability, cognitive impairment, and dementia [3]. The presence of comorbidities complicates the administration of polytherapy and can result in unintended adverse effects. Considering that 12 million Italians use antihypertensive medications, contributing to rising healthcare expenditures for the National Health Service [4], greater attention has been focused on alternative interventions, such as physical exercise. 

The American Heart Association and the American College of Cardiology have recently released updated guidelines that provide broad recommendations for preventing and treating elevated blood pressure through lifestyle approaches [5]. These guidelines emphasize the importance of adopting healthy habits to maintain cardiovascular health [6]. Adopting a healthier lifestyle can have a profound impact on reducing morbidity and the risk of mortality [7,8]. Current guidelines agree on the importance of lifestyle in the treatment and prevention of hypertension, such as dietary patterns and increased physical activity (PA) [9]. Following the 2016 European guidelines on cardiovascular disease prevention and those published by the task force and experts from the European Association of Preventive Cardiology of the European Society of Cardiology [10], the efficacy of exercise training in the prevention and management of cardiovascular diseases is well established, with high levels of PA being associated with a 24–27% lower risk of developing CVD [11]. Overall, the guidelines demonstrated a strong consensus on the significance of increasing PA, with a recommended range of 150 to 300 min of moderate-intensity aerobic exercise [12]. 

Moreover, different types of exercise interventions have been found to be as effective as most antihypertensive drugs in reducing systolic blood pressure (SBP) among adults with HTN [13]. Several meta-analyses have reported that endurance, isometric, and dynamic exercise interventions significantly lower SBP in healthy adults. Despite multiple studies demonstrating the modest but consistent reduction in SBP associated with various exercise interventions across diverse populations and settings, establishing the duration, intensity, and volume of administered exercise is crucial in order to facilitate the required dose-response relationship and recommend effective protocols for efficient interventions. A recent meta-analysis, focused on the effects of exercise regimens and medications on SBP, revealed that the impact of exercise training on SBP remains under-studied, particularly in hypertensive populations, in relation to commonly used antihypertensive drugs [14].

In this randomized clinical trial, our aim was to investigate the impact of a 24-month program of PA, based on a combination of aerobic and resistance training, on antihypertensive drug load among hypertensive subjects without prior cardiovascular diseases (CVDs).

## 2. Materials and Methods

### 2.1. Study Design

This is a single-center, observer-blinded, randomized, parallel-group clinical trial. All participants provided written informed consent, and the study was designed and conducted in accordance with the Declaration of Helsinki and approved by the Ethics Committee of the University of Molise (26119_II/1) and was registered at ClinicalTrials.gov (NCT02236416). 

### 2.2. Participants 

One hundred and eighty community-dwelling volunteers who lead either sedentary or normally active lifestyles [15] were recruited for the study. The participants were selected based on a list of patients with homogeneous severity of hypertension and were essentially undergoing therapy during the same period, as the varying severity of the condition would have necessitated the adjustment of different exercise protocols. The sample characteristics are shown in Table 1. Eighteen individuals were deemed ineligible due to severe clinical conditions, 22 refused to participate, and 10 could not be re-contacted. One hundred and thirty were randomly assigned to either an intervention group (*n* = 65) or a control group (*n* = 65). Of the 130 subjects randomized, 76 participants (23 males and 53 females, with an overall mean age of 66 + 7.917) completed the 24-month assessment. 

**Figure 1:**
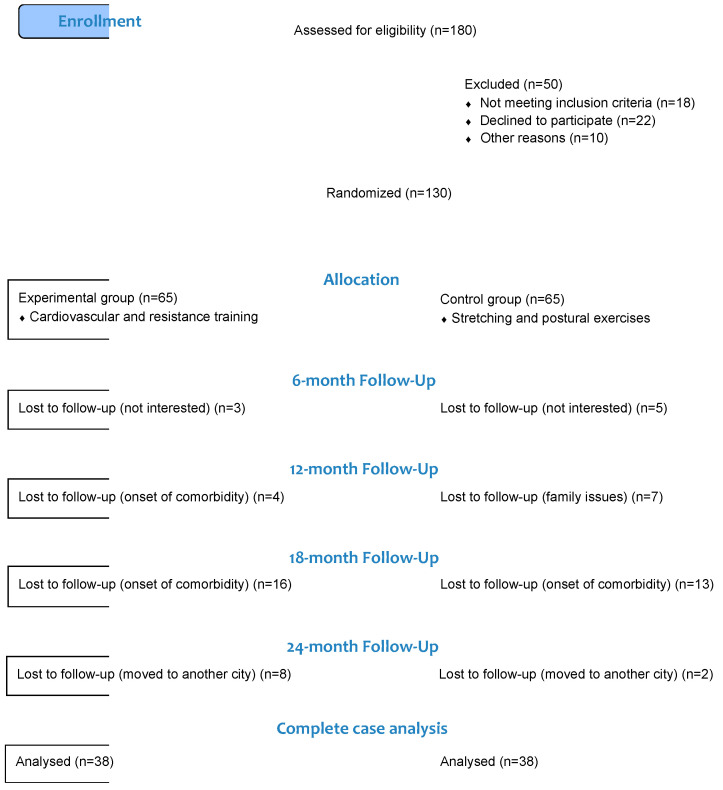
Flowchart of trial profile.

Eligibility criteria included the following: (1) age > 50 years; (2) previous diagnosis of essential HTN; (3) HTN treatment with at least one drug; (4) ability to walk without assistance for 6 min (6-min walking test) [16]. Exclusion criteria included (1) age > 90 years; (2) secondary HTN; (3) systolic office BP ≥ 150 mm Hg and/or diastolic office BP > 90 mm Hg; (4) history of coronary heart disease and/or stroke; (5) presence of high CV risk factors [17]; (6) clinical conditions (neurological, cardio-respiratory, musculoskeletal, etc.) that did not allow for completion of the program of PA; (7) inability to walk without assistance for 6 min (6-min walk test). The flowchart of the study is shown in Figure 1. 

### 2.3. Randomization

The study design was a parallel-group randomized controlled trial. The randomization was conducted following the pre-intervention evaluations, which were conducted by a researcher who was not directly involved in the recruitment or data collection process, using a computer-generated allocation list, in a 1:1 ratio for the experimental and control groups. The allocation list was kept in a sealed envelope. During each evaluation, participants and examiners were explicitly instructed not to disclose or inquire about any information related to the PA program. The EG followed a PA program based on a combination of strength/endurance training, while the CG followed a PA program based on postural/stretching exercises. 

### 2.4. Procedures

#### 2.4.1. Assessment

Individuals who met the eligibility criteria were invited to a face-to-face assessment, during which the physicians involved in the trial collected clinical history, conducted a complete physical examination, and examined laboratory and instrumental tests to evaluate the cardiovascular risk factors. The Physical Activity Scale for the Elderly (PASE) was used to estimate the level of weekly PA [18]. The results of this test allowed the authors to recruit subjects who were homogeneous in terms of lifestyle and levels of weekly physical activity [19]. Antihypertensive drug load was recorded at baseline and at 6, 12, 18, and 24 months after the beginning of treatment. Participants were invited to periodically monitor their BP. If systolic BP values were ≥150 or <100 mm Hg and/or diastolic BP values were >90 or <60 mm Hg, they were invited to consult the attending physician and the cardiologist in order to remodulate the antihypertensive therapy and bring the BP values into the normal range.

#### 2.4.2. Drug Load Estimation

The defined daily dose (DDD) is a statistical measure developed by the World Health Organization (WHO) to assess drug equipotency. It represents the assumed average maintenance dose per day for a drug when used for its primary indication in adults [20]. The prescribed daily dose (PDD) is a measure of the average daily amount of a drug that is actually prescribed. WHO defines it as the average dose prescribed based on a representative sample of prescriptions [20]. To determine the usage of antihypertensive medications, the PDD/DDD ratio is calculated. This ratio indicates the number of DDDs used by an individual patient per day. Specifically, the PDD/DDD ratio was calculated for various classes of medications including diuretics (such as thiazides, loop diuretics, and potassium-sparing diuretics), beta-receptor blockers, calcium antagonists, angiotensin-converting enzyme (ACE) inhibitors, angiotensin receptor blockers, and alpha-receptor blockers [21]. To assess the overall drug load, the sum of the PDD/DDD ratios for all antihypertensive drugs taken by each participant was calculated.

### 2.5. Experimental Training Protocol

The exercise training program used a combination of aerobic and resistance training, with participants performing 30 min of aerobic exercises followed by 20 min of resistance exercises in each session. Each session included a 5-min warm-up and a 5-min cool-down period. Aerobic training was performed using a treadmill or stationary bike, with exercise intensity kept at a moderate level by ensuring that participants did not exceed 65% to 75% of their maximum heart rate (calculated as 220 minus their age). Participants’ exercise sessions were recorded using Polar heart monitors (Polar M430 model). However, it should be noted that relying solely on heart rate to gauge exercise intensity can lead to overestimation of the actual intensity level [22]. Therefore, the Perceived Exertion Scale-RPE [23] was also used to monitor the level of exertion experienced by participants during each intervention session, with ratings ranging from 6 to 20.

In the resistance training intervention, bodyweight exercises were performed along with the incorporation of dumbbells to achieve progressive overload. The aim of this intervention was to target and train all major muscle groups. The exercise sessions were scheduled to take place twice/thrice a week on non-consecutive days, with a minimum of 48 h between sessions. To ensure the safety and adherence of participants, the resistance training was carefully guided and closely supervised by knowledgeable sport scientists with extensive experience in the field. The resistance training session consisted of six dynamic exercises, which were performed in a slow and controlled manner. Each exercise targeted one of the six major muscle groups (legs, back, abdomen, chest, shoulders, and arms).

After a familiarization period, during which participants mastered the correct technique for each exercise, the protocol was established as follows: the number of repetitions for each exercise remained constant at 15 repetitions. However, the exercise volume was gradually increased by adding more sets. Initially, participants performed one set, and over time, this was progressed to a total of three sets. Moreover, the intensity progression was based on the 1 repetition maximum (1 RM), starting from 50% in the 1st week and reaching 80% in the 24th week. As the participants progressed and were able to complete more than 15 repetitions without experiencing moderate fatigue in two consecutive sets, the intensity was increased accordingly. Additionally, resistance levels were individually adjusted to achieve a score of 12–13 on the Borg scale. The resistance program was divided into two phases: the first phase involved 16 weeks of training twice per week, followed by the second phase, which consisted of training three times per week. At the end of each training session, participants engaged in stretching exercises as a cool-down phase. Throughout the entire training session, participants were provided with a heart rate monitor for continuous monitoring of heart rate. Participants were advised not to engage in any other form of supervised physical activity apart from what was proposed by the two study protocols.

### 2.6. Control Group

The PA program consisted of stretching exercises targeting the major joints of the body followed by postural exercises. These sessions were conducted twice a week for the first 8 weeks, followed by an increase to three times per week. The stretching routine included various exercises such as hamstring, soleus, shoulder, and triceps stretches, a standing quadriceps stretch, a lunge with a leg on a chair, and a seated knee-to-chest stretch. The participants were instructed to perform the exercises in a manner that avoided any painful sensations. For each joint, two stretches lasting approximately 30 s each were performed during the sessions. The overall stretching session had a duration of about 30 to 40 min. Additionally, twenty minutes of each session were dedicated to improving posture. This included exercises to address neck and pelvic retroversion, strengthen the core muscles, and practice diaphragmatic breathing techniques. Participants were advised not to engage in any other form of supervised physical activity apart from what was proposed by the two study protocols.

### 2.7. Adherence Assessment 

During the 24-month PA program, participants belonging to the experimental group were expected to attend 256 sessions, which spanned over one year of intervention. Adherence to the PA program was calculated by dividing the number of sessions attended by all participants by the maximum number of possible sessions and multiplying it by 100. Personal trainers recorded the names of participants present at each PA session. Another variable used to assess adherence was the number of follow-up visits participants attended. Each participant was invited to five medical visits throughout the 24 months, including the baseline evaluation. The index visit was determined by dividing the total number of visits during the 24-month intervention period by the maximum number of possible visits within the same period and multiplying it by 100.

### 2.8. Sample Size Calculation

The sample size was established empirically, as no studies on the effects of PA on antihypertensive drug load have been published to date to our knowledge. 

Sample size was calculated using G∗Power (version 3.1.9.6; written by Franz Faul, University of Kiel, Kiel Germany). The following design specifications were considered: test family = *F* tests; statistical test = analysis of variance (ANOVA) repeated measures between factors; α = 0.05; (1–β) = 0.95; effect size *f* = 0.8; number of groups = 2; number of measurements = 5. Sample size estimation indicated 16 participants with a critical *F* value of 4.600.

### 2.9. Statistical Analysis 

Data were analyzed using the SPSS (V. 17.0) statistical software package (SPSS Inc., Chicago, IL, USA). Variables were examined for outliers and extreme values by means of box and normal quantile-quantile plots and Shapiro-Wilk’s and Kolmogorov-Smirnov’s tests. When normal distribution could not be accepted, variable transformations (square, square root, logarithmic, reciprocal of square root or reciprocal transformations) were reviewed. The square root of dependent variables (drug load) helped to improve the distribution shape. Repeated measures analysis of variance (RM-ANOVA) was used to evaluate differences among drug load estimation as the dependent variable and groups (experimental group vs. control group) as the independent variables. The significance level was set at 0.05.

## 3. Results

At baseline, the two groups were homogeneous in terms of age (*p* = 0.063), diastolic (*p* = 0.922) and systolic (*p* = 0.646) blood pressure, and antihypertensive drug load (*p* = 0.425) (Table 1). 

By the end of the study, adherence to the PA program ranged between 75% and 80%, indicating that participants consistently attended a significant portion of the sessions. The index visit, representing the frequency of follow-up visits, was approximately 65%.

**Figure 2:**
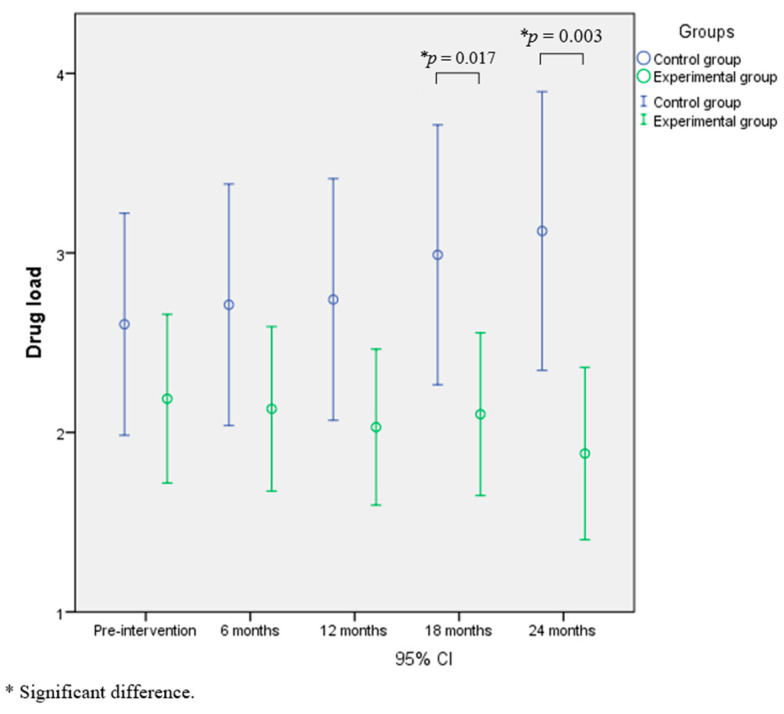
The effect of exercise on drug load.

The RM-ANOVA performed on antihypertensive drug load showed no significant result for time (F_(4,71)_ = 0.84; *p* = 0.506; *n*^2^*p* = 0.045), a significant interaction time×group (F_(4,71)_ = 4.28; *p* = 0.004; *n*^2^*p* = 0.194), and a significant difference between groups (F_(1,74)_ = 3.74; *p* = 0.05; *n*^2^*p* = 0.048). Indeed, a progressive decrease in drug load was observed in the experimental group from the baseline to the 24-month assessment (from 2.24 ± 1.41 to 1.80 ± 1.38; not statistically significant) and a progressive increase in the control group (from 2.54 ± 1.89 to 3.17 ± 2.35; not significant), while the experimental group reported significant low drug load compared to the control group at 18-month (*p* = 0.017) and 24-month assessments (*p* = 0.003). These results are shown in Figure 2.

## 4. Discussion

This study provided evidence that long-term physical exercise incorporating both aerobic and resistance training effectively reduced the dependence on medication in hypertensive patients. The study revealed significant effects starting at 18 months, which became even more pronounced after 24 months (Figure 1). Therefore, to enhance hypertension control, a long duration of physical exercise application including aerobic and resistance training is needed. Moreover, the second guarantee of the intervention’s success is the high adherence to intervention protocols, as already demonstrated by our team in a previous study [24]. 

PA prescription is highly recommended as an effective treatment option for all patients, including those with mild to moderate risk of elevated blood pressure [25]. Several studies have evaluated the role of exercise on SBP, DBP, and HR reduction [26,27,28], indicating that improving awareness and adherence of hypertensive patients to a healthier lifestyle, which includes dietary modifications, stress management, and PA, resulted in successful blood pressure control and enhanced response to drug therapy [29,30]. 

While the potential of replacing pharmacological methods for BP control with lifestyle strategies has been widely theorized, there is no unanimous consensus on the level of efficacy of the different types and modalities of PA on pressure control. Comparing the results of different studies is challenging due to the variations not only in the characteristics of populations analyzed but also in the interventions that encompass a wide range of exercise modalities in terms of frequency, intensity, duration, and formulation, as indicated by a recent meta-analysis [14]. Conversely, corrected guidelines provide clinicians with clear instructions on how to assess, prescribe, counsel, and refer patients to promote increased PA [31]. By following these guidelines, clinicians can play a crucial role in supporting and encouraging their patients to engage in regular PA and to reduce reliance on medications in order to control systolic and diastolic blood pressure levels. 

Aerobic exercise proves to be an effective strategy for improving blood pressure control in patients undergoing pharmacological treatment. However, it is important that the exercise intensity falls within the moderate to vigorous range and that the duration is maintained for a minimum of three to four months. The recommended frequency is three times per week, with each session lasting at least 40–45 min, to achieve the desired effectiveness [32,33].

Several studies have indicated that strength training also holds therapeutic potential for managing arterial hypertension by significantly reducing both systolic blood pressure (SBP) and diastolic blood pressure (DBP), especially when conducted with moderate to vigorous load intensity [34,35], whereas Ribeiro Correia [32], in a recent meta-analysis, highlighted that SBP has greater sensitivity to strength training compared to DBP. According to a 2016 review conducted by Sabbahi [36], both aerobic and resistance training were found to have a comparable effect in reducing blood pressure. However, it is worth noting that the underlying physiological mechanisms may differ between the two types of training. Based on these findings, the optimal approach to enhancing the health of older adults with chronic heart problems and hypertension is concurrent training, which integrates both strength and aerobic exercises at a moderate to vigorous load intensity. This approach, as adopted in long-term interventions such as in the current study, has been supported by recent research [37,38]. The choice to use combined aerobic and resistance training had a dual purpose: firstly, to ensure that participants experienced similar effects on both SBP and DBP reduction through moderate and varied activities and secondly, to provide muscle strengthening that could promote the safety of elderly individuals in their daily activities and social life. 

Moreover, establishing the frequency, intensity, and volume of supervised PA is essential for achieving improvements in both SBP and DBP. 

Frequency in performing PA, up to incorporating regular PA into a daily routine, can significantly improve cardiovascular health and reduce the risk of complications associated with high blood pressure and cholesterol [39]. 

The impact of exercise can vary depending on the intensity and volume employed. Intensity is related to low risk of hypertension [40]. Epidemiological studies indicate that higher intensity exercise can effectively lower blood pressure, while excessive intensity may have negative effects on health. Therefore, we established a moderate-intensity training intervention that is considered the optimal choice for reducing systolic blood pressure (SBP). Additionally, for diastolic blood pressure (DBP) reduction, moderate-intensity training remains the most favorable option, as suggested by several studies [41,42].

In terms of volume, aerobic interventions involving high-volume exercise have been shown to contribute to blood pressure reduction [28,43]. However, a previous study, comparing inactive individuals with those engaging in free walking activities (characterized by very low intensity but higher volume of physical activity) did not find significant differences in SBP changes [44]. Therefore, a volume of exercise without sufficient intensity does not provide adequate stimulation to the body for blood pressure control. 

Cornelissen and colleagues [26] showed that the volume of strength training is more effective than other load characteristics such as weekly frequency to reduce blood pressure. We can deduce that the optimal combination of intensity and volume has ensured the best effects on blood pressure control and consequently on reducing the need for medication in our sample. 

In conclusion, there are three essential conditions that must be considered when designing a physical protocol with the goal of reducing blood pressure and, consequently, medication intake: a long-term commitment to regular PA; good adherence to the exercise protocol; and appropriate volume, intensity, and modality of exercise [28]. Enhancing enjoyment and motivation through various strategies, such as group exercise or the utilization of technological equipment, could facilitate long-term adherence to physical activity programs for the elderly [45,46].

The trend of improvement regarding the drug load intake in the EG and the trend of drug load increase in the CG, although not significant over time, results in a significant difference between the groups at 18 months and even more so at 24 months (confirmed by significant interaction time*group). This trend certifies the protective value of physical exercise against the aging process and its related health risk factors.

However, it is important to note that our research findings cannot be directly compared to similar studies because, to our knowledge, we uniquely considered drug load as the outcome measure. Anyway, our data further support the significant role of PA in replacing or at least reducing blood pressure-lowering drugs and encourage clinicians to strongly recommend that their patients engage in at least of 30–45 min of moderate-intensity physical exercise, as suggested by International Guidelines [47]. 

## 5. Limitations

The study did not include lifestyle modifications such as dietary changes, smoking cessation, or weight loss, which could have potentially impacted the results. However, it should be noted that participants were instructed to maintain their usual habits throughout the duration of the study. 

Adherence in the study was assessed not in terms of dropouts but rather in terms of the number of sessions and medical check-ups scheduled for and attended by the participants to ensure the intervention’s results [48].

Moreover, the loss of some follow-up assessments could have influenced the magnitude of benefits observed in the experimental group and potentially underestimated the presumed disadvantage in the control group participants. These losses may be attributed to the lengthy duration of our study, which spanned 24 months, requiring a significant commitment from the participants.

Finally, it should be mentioned that due to the high proportion of women in our sample population, there is a potential for gender bias. However, our analyses demonstrated that there were no significant differences in effectiveness based on gender.

## Figures and Tables

**Table 1:** Sample characteristics. Data are presented as mean and standard deviation (SD).

Variables	Control Group	Experimental Group	*p*-Value
Age (years)	68.00 ± 8.33	64.67 ± 7.31	0.063
SBP (mmHg)	136.79 ± 8.15	136.49 ± 8.24	0.870
DBP (mmHg)	84.49 ± 5.94	84.32 ± 6.25	0.908
Drug Load (PDD/DDD)	2.60 ± 1.88	2.22 ± 1.42	0.425
PASE	93.98 ± 49.72	99.23 ± 45.69	0.620

SBP: Systolic blood pressure; DBP: Diastolic blood pressure; PDD: Prescribed daily dose; DDD: Defined daily dose; PASE: Physical activity scale for the elderly.

## Data Availability

The data presented in this study are available upon request from the corresponding author.
